# DNA Dynamics Is Likely to Be a Factor in the Genomic Nucleotide Repeats Expansions Related to Diseases

**DOI:** 10.1371/journal.pone.0019800

**Published:** 2011-05-20

**Authors:** Boian S. Alexandrov, Vlad I. Valtchinov, Ludmil B. Alexandrov, Vladimir Gelev, Yossi Dagon, Jonathan Bock, Isaac S. Kohane, Kim Ø. Rasmussen, Alan R. Bishop, Anny Usheva

**Affiliations:** 1 Theoretical Division, Los Alamos National Laboratory, Los Alamos, New Mexico, United States of America; 2 National Center for Biomedical Computing, Informatics for Integrating Biology and the Bedside, Boston, Massachusetts, United States of America; 3 Endocrinology, Beth Israel Deaconess Medical Center, Harvard Medical School, Boston, Massachusetts, United States of America; Newcastle University, United Kingdom

## Abstract

Trinucleotide repeats sequences (TRS) represent a common type of genomic DNA motif whose expansion is associated with a large number of human diseases. The driving molecular mechanisms of the TRS ongoing dynamic expansion across generations and within tissues and its influence on genomic DNA functions are not well understood. Here we report results for a novel and notable collective breathing behavior of genomic DNA of tandem TRS, leading to propensity for large local DNA transient openings at physiological temperature. Our Langevin molecular dynamics (LMD) and Markov Chain Monte Carlo (MCMC) simulations demonstrate that the patterns of openings of various TRSs depend specifically on their length. The collective propensity for DNA strand separation of repeated sequences serves as a precursor for outsized intermediate bubble states independently of the G/C-content. We report that repeats have the potential to interfere with the binding of transcription factors to their consensus sequence by altered DNA breathing dynamics in proximity of the binding sites. These observations might influence ongoing attempts to use LMD and MCMC simulations for TRS–related modeling of genomic DNA functionality in elucidating the common denominators of the dynamic TRS expansion mutation with potential therapeutic applications.

## Introduction

Repetitive DNA sequence elements are widely abundant in the human and the other eukaryotic genomes. They are classified into two large families, the “tandem” and “dispersed” repeats. The trinucleotide repeats sequences (TRS) represent the most common type of tandem microsatellites in the vertebrate genomic DNA. Such genomic elements were found in the coding and the noncoding DNA co-localizing with human chromosomal fragile sites that are associated with genomic breakpoints in cancer and a growing number of devastating human diseases [1], [2], [3], [4], [5]. TRS disorders typically have large and variable repeat expansions [6] that result in multiple tissue dysfunction or degeneration. The neurological disorder Friedreich's ataxia (FRDA) co insides with expansion of a genetically unstable (GAA· TTC)_N_ tract in the first intron of the frataxin gene [7], [8], [9] resulting in the transcriptional inhibition of the gene. The (CTG.CAG)_N_ repeats in the Huntington's disorder (HD) is one of the most highly variable TRS in the human population [10], [11]. In the fragile X syndrome (FXS) the (CGG.GCC) expansion in the 5′ untranslated region of the FMR1 gene causes the transcriptional silencing of the gene [12].

The expression of fragility was found to be dependent upon the TRS expansion beyond a threshold of copies in tandem. DNA replication, transcription and DNA repair are important cis-acting factors in the process of TRS amplification [13], [14], [15], [16]. The exact mechanisms that drive expansion and the TRS specific expansion effect on genomic DNA functions are presently not well understood.

It is commonly accepted that the TRS amplification cause formation of non B-DNA structures that could disrupt normal cellular processes [17], [18]. The formation of such structures starts with transient DNA openings, i.e. local DNA melting and bubbles [19] that extend from a few to a hundreds of DNA base pairs. Experimental results with A/T-reach repeats reveals that their expansion is usually initiated with transient local DNA melting (bubble formation) that could next extend into static loops or non-B-DNA structures [16], [17], [18]. Our recent sequence specific breathing DNA dynamics observations suggest that transient DNA bubbles form not only in A/T-reach sequences but also in sequences with relatively high G/C-content caused by the softness of the base pair stacking [20]–[23]. Therefore, transient DNA bubbles is expected to form in the G/C-reach (CTG.CAG)n and (CGG.CCG)n TRSs as well as in the (GAA.TTC)n sequences with high A/T-content. It is likely that the local base pair dynamics may display some sequence and number of repeats specificity that could underline the propensity for expansion and possibly alteration in genomic DNA functions. Local bubble formations that extends from a few to several base pairs could shift from stable to more unstable structures that interact with nuclear components promoting further TRS expansion.

Using the concept of “intermediate bubble states” and our recently established criterion for DNA base pair “thickness” through the base pairs average displacement (BAD) characteristic [22], we compare the breathing dynamics of TRS against random sequences with identical nucleotide composition as well as repeats with different lengths and G/C content. We report results for a notable coherent dynamical behavior of the TRS, leading to an enhanced tendency for forming large and stable local DNA-opening modes at physiological temperatures. The synchronized behavior of the average displacements from the equilibrium positions of the base-pairs in TRS is suggestive of a possible advance of extended intermediate states that are known to be strong precursors for transient bubble formation. Our LMD and MCMC simulations of TRS with different G/C content and number of repeats demonstrate appearance of large transient bubbles that depend on the TRS length. We provide an experimental example of how the TRS bubble spectrum could interfere with protein-DNA interactions. We specifically demonstrate that the flanking TRS has a profound effect on the spectrum of the TATA-box DNA dynamic activity that could explain the lost TFIID-TATA binding. We propose that presence of repeats in the noncoding genomic DNA could nucleate the formation of bubbles that directly interfere with specific gene expression by altering protein-DNA binding [12]. Our findings could shed some light and facilitate functional predictions of the effect of TRS expansions on gene expression.

## Results

### Expansion of repeats leads to well-pronounced synchronized DNA breathing behavior

To elucidate the effect of repeats' amplification on the DNA breathing behavior we compared the local base pair breathing of the frataxin (GAA.TTC)_N_ TRS with varying repeats number [24]. As a measurement we use the BAD criterion, which represents the thermal base pair stability or “softness” due to base pair breathing at physiological temperature and salt content. BADs are directly connected with the DNA melting simulation procedure. In general, DNA denaturation is a ‘close-to-open’ state transition of the double helix. This transition can be visualized by considering the fraction of intact hydrogen bonds between complimentary nucleotides as a function of the temperature. It is well known that when DNA is melting, i.e. opening, the transition is initiated at lower temperatures first in the “soft”, i.e. A/T-rich, DNA regions, where the stabilizing hydrogen bonds are only two per base pair. As the temperature increases, the mixed A/T-G/C regions also begin to melt, and finally the G/C-rich regions undergo denaturation. The local openings of the DNA double helix can be measured through the UV absorption as the UV absorption depends on the averaged fraction of the open base pairs in DNA. DNA melting can be simulated by calculation of the average fraction of open DNA base pairs at the given temperature, i.e. by calculating the average DNA base pair displacements, that is the BADs. Here, the BAD profiles [20], [21] are calculated from MCMC simulations based on the EPBD model of DNA dynamics [21], [25].

**Figure 1:**
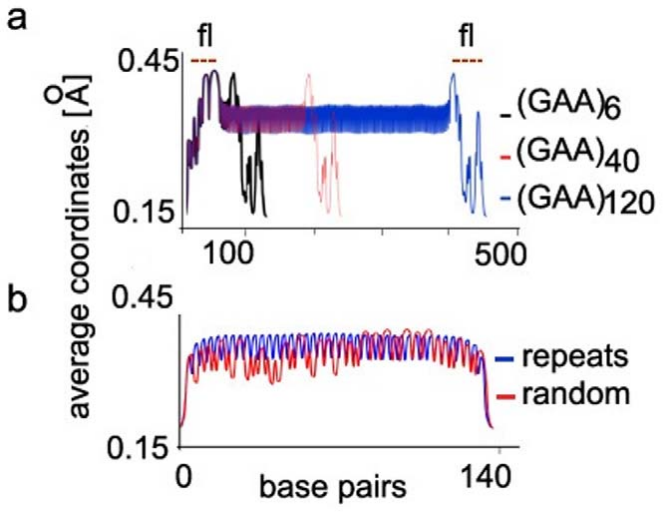
Accumulation of (GAA.TTC) repeats leads to changes in local DNA breathing. BAD criteria are used to describe and compare the local base pair breathing of DNA sequences with different numbers of (GAA.TTC) repeats embedded within the frataxin gene [B7] promoter sequence. a) BAD coordinates [Å] are calculated with EPBD based MCMC simulations for sequence inserts with different numbers of repeats: (GAA.TTC)_6_-black line, (GAA.TTC)_45_-red line, and (GAA.TTC)_120_-blue line. The position of the flanking sequence (fl) is shown above the panel. b) BAD coordinates for a randomized sequence with the same number of base pairs and G+C content as the (GAA.TTC)_41_ sequence. The random sequence (red line) is missing the synchronized average base pair openings behavior of the symmetric (GAA.TTC)_41_ (blue line). The nucleotide position is shown on the horizontal. The BAD coordinates are shown on the vertical in [Å].

The BAD base pair values of (GAA.TTC)_N_ with three different numbers of repeats (N = 6, 40, and 120) are shown in Figure 1. Both, the (GAA.TTC)_40_ and (GAA.TTC)_120_ TRSs demonstrate well pronounced coherent BAD profile, that correlates with the number of repeats (panel a). There is no such coherency in the flanking genomic sequence or in the significantly shorter (GAA.TTC)_6_ TRS.

Moreover, Figure 1b compares the BAD profiles of the repeat sequence (GAA.TTC)41 with a profile arising from a random sequence with the same length and G+A content. While the magnitudes of the BADs are comparable there is a clear lack of coherency in the case of the random sequence. This effect is not specific to the shown sequence but occurs for any non-periodic sequence. This observation of TRS length-dependent synchronized BADs behavior is novel, and the resulting collective behavior of the tandem repeats may serve as a precursor for outsized intermediate bubble states.

### Longer repeats exhibit well pronounced intermediate bubble states

**Figure 2:**
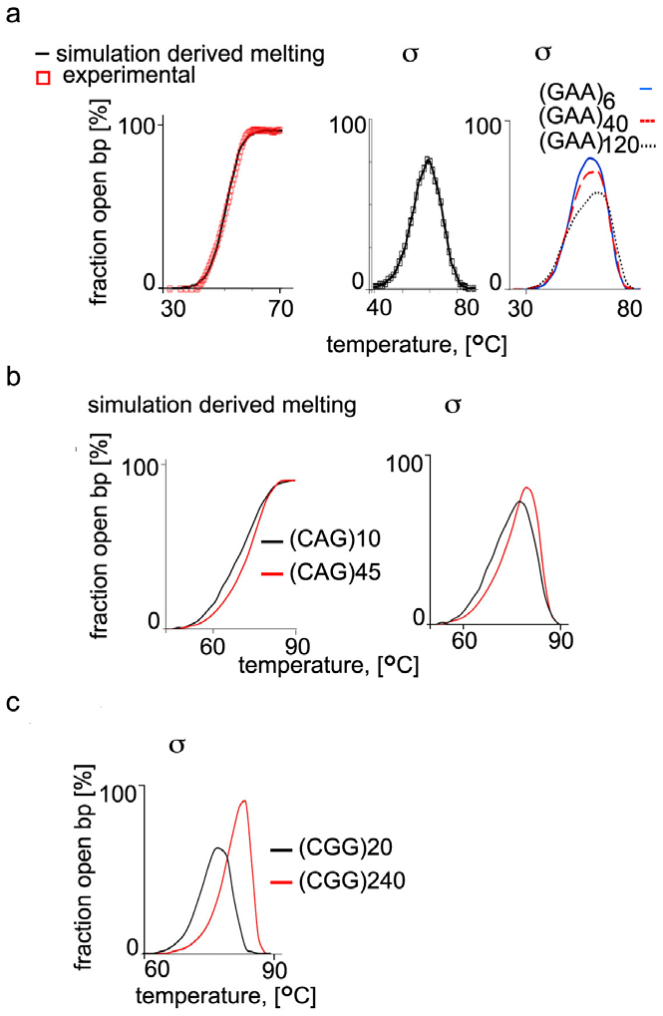
Formation of extended intermediate states in repeated sequences in response to temperature elevation. a) Melting behavior of the (GAA.TTC)_41_ sequence and intermediate bubble state generation. MCMC simulated (black line) and measured (red squares) melting curve for the (GAA.TTC)_41_ sequence at physiological salt concentration (100 mM KCl) is presented on the *left*. The theoretical fraction of the (GAA.TTC)_41_ bases in the intermediate bubble states is calculated as well (panel in the *middle*). The *σ* values for (GAA.TTC)_N_ of various lengths is compared and presented at the *right*. The sequences with a smaller number of repeats have higher, more pronounced *σ* peaks that are shifted toward lower temperature when compared to the sequences with higher number of repeats. The identity of the repeats and the corresponding curves is shown at the top. b) Simulated melting behavior (*left* panel) and intermediate bubble state σ of 6 and 45 (CAG.CTG) repeats (*right* panel). c) The intermediate bubble state **σ** of 20 and 240 (CGG.CCG) repeats. The higher intermediate state is consistent with the collective behavior of the repeats. The σ value is presented on the vertical as a fraction of open base pairs at a certain temperature (horizontal, °C). The total number of base pairs in the simulated individual sequences is taken as 100%.

The synchronized BAD behavior in TRS sequences suggests for possible formation of extended intermediate bubble states at elevated temperatures that are known to be strong precursors for transient bubble formation [20], [21]. The fraction of the open base pairs at higher temperature that is a basic characteristic for the intermediate bubbles states, could differ based on the TRSs sequence and the number of repeats. In Figure 2, panels *a*, left we show results from our MCMC simulations together with experimentally derived, normalized UV-absorption melting curve for (GAA.TTC)_41_ repeats. The experimental melting conditions are described in the Materials and Methods section. The results for the (GAA.TTC)_41_ repeats (panel a) demonstrate an excellent agreement between our simulations and the experimental data.

The EPBD model, which accurately determines the DNA melting behavior [21], could also be applied to derive the parameter σ (Figure 2, panel a, in the middle) that quantifies the intermediate bubble states [22]. The intermediate bubble states of DNA present local permanent openings of DNA at temperatures where the DNA molecules are mainly at a double helix state (not denatured) with only partial permanent openings of the double strands. The parameter σ was previously introduced [22] as a simple experimental and theoretical measure of the *average* size of the intermediate bubbles states. More specifically, σ = f–p; where f - is the average fraction of the open DNA base pairs, and p is the average number of the entirely denatured DNA molecules at the given temperature [25].

We initially compared the *σ* values derived by our EPBD based MCMC simulations for sequences with different numbers of (GAA.TTC)_N_ (N = 6, 40, 120) repeats (Figure 2, panel a, right). The larger and more pronounced peaks for longer repeat segments are clear indications that the dsDNA repeat tracts sustain larger intermediate bubbles. A shifting *σ_max_* values tendency for longer repeat tracks toward lower temperatures is clearly visible for these TRSs, which correspond to a lower melting temperature. Such tendency is not observed for the G/C-reach (CAG.GTC)_N_ (panel b) and (CGG.CCG)_N_ (panel c). For these sequences the *σ_max_* is notably shifted toward higher temperatures for the longer repeats as compared to the shorter TRSs. The shift to higher melting temperatures correlates with the increased TRS G/C content. Importantly, the *σ_max_* value (i.e. the fraction of the open base pairs forming the maximal intermediate bubble) increases with the number of repeats, in the same fashion as the dynamical instability mutations, i.e. accelerating with longer repeats tracts and for generally ‘softer’ repeat sequences.

Interestingly, this acceleration does not exclusively depend on the AT-content, i.e. on the hydrogen bonds-governing “softness” of the DNA sequence. The reason of this behavior is rather in the collective breathing behavior of DNA repeats and the “stacking softness” [21], which triggers their simultaneous opening, although at elevated temperatures for highly GC-rich repeats. The collective breathing behavior of the repeats causes the simultaneous strand separation independently of the high C/G content. To present this more distinctively we plot, in Figure 2, panels *b*, the melting curves and the σ values (as a function of the temperature) for two HD (GAC.GTC)_N = 10, 45_ tracts, as well as the σ values for two FXS (CGG.CCG)_N = 20, 240_ TRSs , in Figure 2 panel c, with their actual left and right flanking genomic sequences.

The results clearly demonstrate that while the melting temperatures increase together with the length of the repeats in both cases (because of the increased GC-content) the maximal intermediate bubble state is becoming more pronounced in the MRSs with higher number of repeats. This means that the maximal fraction of base pairs that open coherently increases with the number of repeats irrespectively of the higher G/C content and the sequence specifics.

### LMD simulations of the local DNA breathing dynamics at the repeat tracks

The length of the TRSs influences the lifetimes of the local transient DNA bubbles in the repeat tract, i.e. it directly influences the local DNA breathing dynamics. The TRSs length dependent BAD behavior could also display an effect locally on the intermediate base pairs states [21], [25] i.e. the lifetime of the local bubbles. We applied our LMD simulations [20] to derive this effect and compare it for TRSs with different A/T, G/C content, and number of repeats.

**Figure 3:**
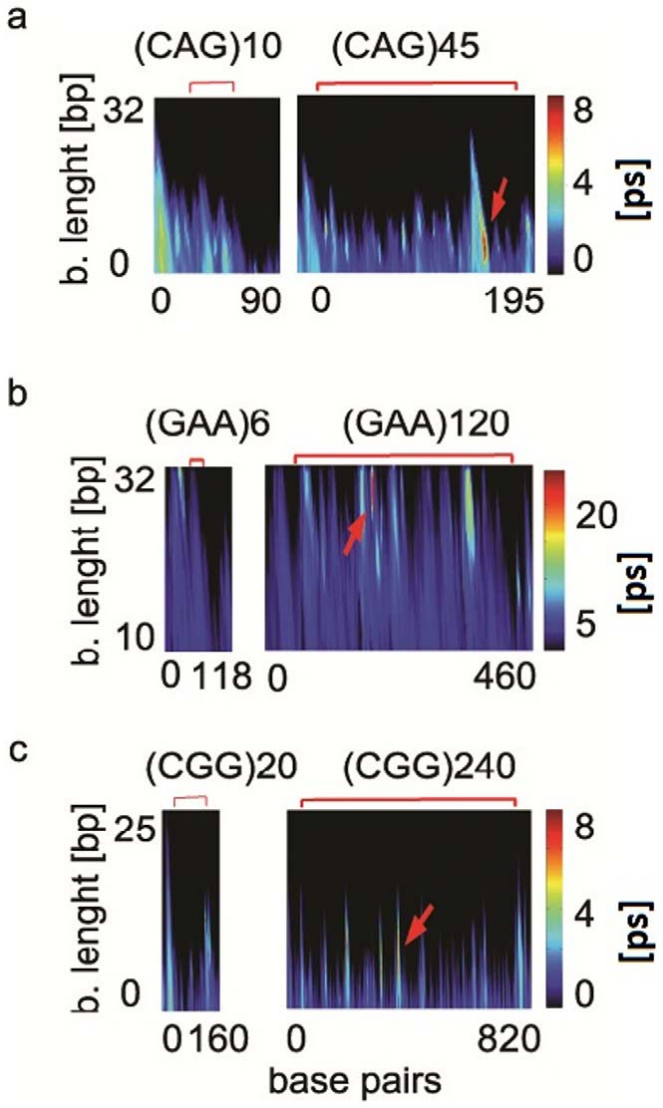
The TRS expansion has an effect on the DNA bubble spectrum. EPBD based LMD simulations have been conducted on the: a) (CAG.CTG)_45_ repeats and healthy (CAG.CTG)_10_ repeats with 30 bp flanking huntington gene sequence; b) (GAA.TTC)_120_ and (GAA.TTC)_6_ MRS that are embedded in 50 bp frataxin gene sequence; c) (CGG.GCC)_240_ and (CGG.GCC)_20_ repeats together with 50 bp FMR1 gene flanking sequence. The y-axis represents the length of the bubbles in bp; the x-axis represents the number of the base pairs; the color axis gives the bubble duration in psec. The brackets above the panels denote the repeated sequence; red arrows- the largest and long-lived base-pairs openings.

We conducted EPBD-based LMD simulations (Figure 3) on the following TRSs: (CAG.CTG)_10_ and (CAG.CTG)_45_ with their actual flanking sequences in the Huntington gene (panel a); (GAA.TTC)_6_ and (GAA.TTC)_120_ with the frataxin gene flanking (panel b) ; (CGG.CCG)_20_ and (CGG.CCG)_240_ respectively with the FMR1 gene flanking sequences (panel c). The simulation data demonstrates well-pronounced appearance of large and long-lived DNA local base pairs openings in the longer TRSs. The (GAA.TTC)_120,_ (CAG.CTG)_45_, and (CGG.CCG)_240_ dynamical patterns clearly demonstrate that the TRS expansion is shaping the lifetimes of the bubbles. The large and long-lived base-pairs openings in the (GAA.TTC)_120_ center are at least twice as high as for the shorter (GAA.TTC)_6_ TRSs. This tendency is present in the (CAG.CTG)_45_, and (CGG.CCG)_240_ TRSs as well. Although both, the long and the shorter TRSs have identical flanking sequences such kind of long-lived large openings lack in the short repeat tracts.

The above data indicates that the repeat expansion coincides with significant changes in the local DNA breathing dynamics. The appearance of specific features of the bubble spectrum, viz. long lived large bubbles is profoundly influenced by the size of the repeated sequence.

### Repeats interfere with the function of transcription factors binding sites by altered local DNA breathing dynamics

The observed activities are striking manifestation of how accumulation of repeats could have a profound effect on the local DNA breathing dynamics. The transient collective openings in the double helix due to TRSs in the noncoding genomic DNA may seed the formation of bubbles that could interfere with DNA-protein interactions involved in a gene specific transcriptional regulation [12]. This notion is supported by the experimental observation that the presence of a certain number of repeats could promote cellular protein-DNA binding [26].

**Figure 4:**
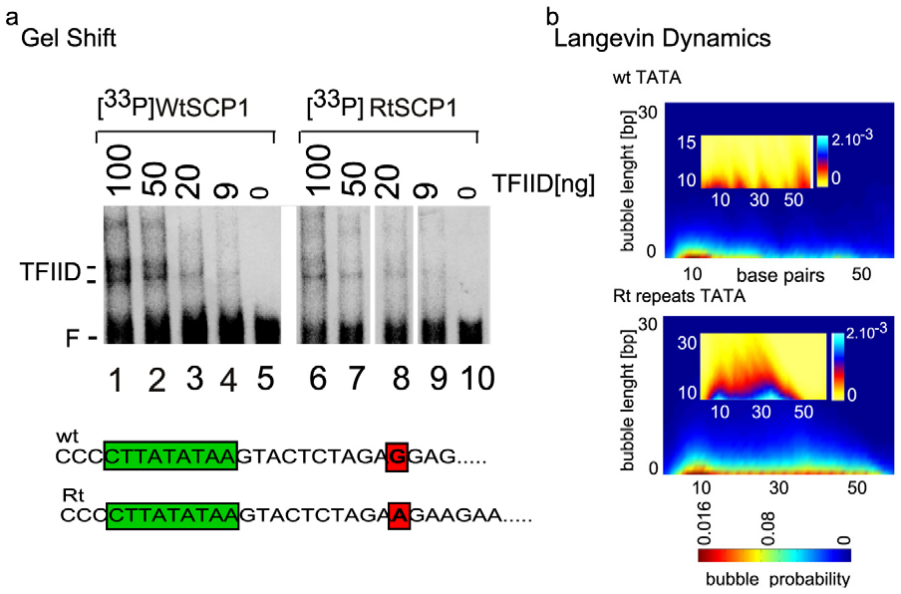
Effect of TRS on TFIID-TATA box DNA binding and local DNA dynamics. a) Band shift titration reactions received [^33^P]-labeled, double-stranded oligonucleotides containing the 60 bp long SCP1 TATA box sequence (10 nm): RtSCP1-mutant SCP1 variant contains 15 (GAA.TTC) repeats downstream of the TATA box; Wt SCP1-wild type SCP1 promoter sequence as indicated at the top of the panel. All reactions are conducted at 370C and identical buffer conditions. The reactions in lanes 1–4 and 6–9 received different amount of purified human TFIID complex as indicated at the top of the lanes. The reactions in lanes 5 and 10 did not receive TFIID. The positions of the free DNA (F), and TFIID complexes (TFIID) are indicated on the right. The sequence of the Wt SCP1 oligo and the mutant Rt SCP1 is shown below the panel. The TATA box sequence is underlined. b) LMDs simulations of the collective DNA openings in Wt SCP1 and RtSCP1 sequences. The top panel- wSCP1 bubble probability; bottom panel- Rt SCP1 bubble probability. The insets (yellow) -the probabilities (colored axis on the left: 0–2.10^−3^) for bubbles over 10 bp; y-axis - length of the bubbles in bp; x-axis - number of the base pair.

We used a TATA box gene promoter sequence (SCP1) [27] as a test case for the general transcription factor complex TFIID-TATA box DNA binding in the presence of (GAA.TTC)_15_ TRS immediately downstream of the wild type intact TATA box sequence (Figure 4). To probe for TFIID binding to such TATA-TRS flanking variant sequence (RtSCP1), we performed gel shift experiments with a purified from HeLa cells TFIID protein complex (panel b) [28]. As a positive control, we conducted gel shift reactions with the wilt type promoter fragment (Wt SCP1). Reactions were assembled with equal protein amounts of TFIID. The results clearly suggest that the RtSCP1 binds TFIID less tightly compared to WtSCP1 and 100 ng and 50 ng of protein shifts significantly less WtSCP1 (compare lanes 1, 2, 3 with lanes 6, 7, 8). This result demonstrates that the presence of (GAA.TTC)_15_ repeats leads to inhibition of TATA box-TFIID binding although the TATA sequence is entirely preserved. The LMD simulations reveal that the bubble spectrum of the wild type promoter is significantly altered when the TATA-box flanking sequence is replaced by (GAA.TTC)_15_ (panel b) although the replacement does not disturb any of the TFIID-TATA DNA point of contacts [29].

The LMD simulation predicts that the flanking TRS has a profound effect on the spectrum of the local TATA-box dynamic activity that significantly differs from the TATA spectrum in the wild type flanking sequence environment. Importantly, this prediction also coincides with the absence of TFIID binding to the TATA-TRS flanking oligonucleotide. Although the repeats do not disturb any of the TFIID points of contact [29] the altered local TATA box dynamics could explain the loss of TFIID binding.

## Discussion

We report a novel coherent DNA breathing behavior in TRSs that is readily calculated using the EPBD derived values of the base pairs average displacements. We describe a synchronized BADs behavior that clearly depends on the length of the TRSs. The expansion of repeats results in a measurable collective TRS specific breathing dynamics. The collective behavior leads to the appearance of significantly enhanced DNA intermediate bubble states when compared to sequences with a random nucleotide composition or with much shorter repeat tracts. We propose that the collective propensity of TRSs breathing could serve as a precursor for overextended intermediate bubble length and life-times. Similar behaviors have been previously reported for A/T-rich repeats sequences, but not in G/C reach TRSs [30].

The correlation between repeats expansion and DNA “stacking softness” is quantified by the calculated value of the intermediate bubble state parameter σ. The value of this parameter correlates to the experimentally determined DNA melting values and size of the intermediate bubbles [22] that are directly related to the DNA breathing dynamics. Our observation is that the σ_max_ increases with the number of repeats and independently of the A/T content of the TRS. The effect corresponds to the collective BADs behavior and it is likely to be caused by the TRS periodicity. Such striking result connects the average TRSs behavior, BADs, and maximal intermediate bubble states independently of the A/T- content. It is likely that the TRS expansion in the disease-related sequences could lead to enhanced coherent DNA openings i.e. enhanced local strand separations when compared to the “healthy” sequences with a low number of repeats. This could explain at least in part, the previously described tendency of sequences with a larger number of repeats to form uncommon non-B DNA structure conformations [15].

The DNA bubble spectrum, calculated by LMD simulations, also reveals TRS length-related profile of transient bubbles appearance. Based on findings by other groups and the reported here protein-DNA binding results one could expect that the amplification of repeats might nucleate transient bubbles that selectively alter binding of proteins involved in repeats expansion while preventing binding of expansion inhibitors [31]–[33]. Furthermore, TRSs expansion and bubble nucleation in the noncoding genomic DNA might alter binding of transcription factors [28] resulting in alterations of specific gene expression [34], [35]. Our TFIID-TATA box binding data together with the recently published observation by Kunicki group [26] directly support such notion.

The correlation between the transient bubble spectrum and repeats expansion in the individual genomes and gene regulatory sequences could be considered as a local DNA dynamics “epigenetic” determinant. The proposed novel dynamic-related role of repeat expansion in the genomic DNA functionality has far reaching implications for interpretation of genomic data in health and disease.

## Materials and Methods

### Computer simulations

The EPBD model is an extension of the classical Peyrard-Bishop-Dauxois nonlinear model [36] that includes inhomogeneous stacking potential [22]. The LMD and MCMC computer simulations are based on the EPBD model [22] as previously described [20], [21]. It is important to note that both simulation methods are used to generate equilibrium quantities. The LMD generates a number of trajectories the average over which allows the determination of temporal information such as averaged bubble duration etc. The MCMC method does not offer access to temporal information but is computationally much faster. The simulated sequences are with the genomic flanks for: frataxin gene (GAA.TTC)_N_ repeats: N = 6, 40, and 120, ACATGGTGAAACCCAGTATCTACTAAAAAATACAAA AAAAA AAAAAAAA(GAA)_N_ AAATAAAGAAAAGTTAGCCGGGCGTGG TGTCGCGCGCCT GTAATCCCAGC; huntington (CAG)_N_ repeats: N = 10, and 45: ATGAAGGCCTT CGAGTCCCTCAAGTCCTTC(CAG)_N_ CAACAGCCGCCACCGCCGCCGC CGCCGCCGC; FMR1 gene (CGG)_N_ repeats: N = 20, and 240: CGGGCGGCGGCGGTGACGGAGGCGCCGCTGCCAGGGGGCGTGCG GCAGCG(CGG)_N_ CTGGGCCTCGAGCGCCCGCAGCCCACCTCTCGGGG GCGGGCTCCCGGCGC. All simulation are performed at T = 37°C.

### Base pair Average displacement (BAD)

BAD is a new criterion that has been previously introduced to describe the local base pair breathing dynamics [20], [21]. It represents an average characteristic of DNA breathing, viz. BADs are the average displacement of the nucleotides from their equilibrium positions. BADs are calculated with the MCMC techniques, and the results are equivalent to those derived from LMD simulations [20].

### Gel shift reactions

Gel shift reactions are assembled with 20 ng of purified TFIID complex from HeLa cells as previously described [28]. The SCP1 promoter fragment sequence is as in [27]. The sequences of the oligos that have been used in the reactions are: Wt SCP1-CGCCCTTATATAAGTACTC TAGAGGATCCC CGGGT ACC GAGCTCGAATTCA CTGGCCGTCGGCG; RtSCP1-CGCCCTTATATAAGTA (GAA)_15_ GCG.

### DNA melting curve

All DNA oligos were synthesized and gel- and HPLC-purified at the Midland Certified DNA Synthesis Facility, and further characterized for melting behavior as previously described [20]. The DNA was dissolved to 200 mM in 30 mM K phosphate buffer pH 7.5, 100 mM KCl, 1 mM EDTA. dsDNA melting curves were collected for 20°C–105°C at 250–280 nm on a Varian Cary 50 Bio UV/Vis spectrometer equipped with a Peltier probe. Melting data were collected from five independent experiments. The DNA oligonucleotide sequence using in the melting experiments is: CGCG (GAA.CTT)_41_ CGCG.
