# Correction: DNA Dynamics Is Likely to Be a Factor in the Genomic Nucleotide Repeats Expansions Related to Diseases

**DOI:** 10.1371/annotation/f68e9b71-cae1-4f9c-9308-a444d3ea753d

**Published:** 2011-06-22

**Authors:** Boian S. Alexandrov, Vlad I. Valtchinov, Ludmil B. Alexandrov, Vladimir Gelev, Yossi Dagon, Jonathan Bock, Isaac S. Kohane, Kim Ø. Rasmussen, Alan R. Bishop, Anny Usheva

A funding source is missing from the financial disclosure section. The authors wish to add the following to their funding information: "The research of ISK and VIV was supported by a grant from National Library of Medicine (ARRA) R01 LM010125."

